# Arterial Branching Patterns Supplying the Left Upper Lobe of the Lung and Their Incidence: A Systematic Review

**DOI:** 10.3390/jcm15051724

**Published:** 2026-02-25

**Authors:** Kamil Jacek Dworski, Michał Tulski, Sławomir Woźniak, Maria Anders, Chao-An Kuo, Renata Taboła

**Affiliations:** 1 Department of Anatomy, Wrocław Medical University, Tytusa Chalubinskiego 6a, 50-368 Wrocław, Poland; kamil.dworski@student.umw.edu.pl (K.J.D.); michal.tulski@student.umw.edu.pl (M.T.); slawomir.wozniak@umw.edu.pl (S.W.); kcakca29@gmail.com (C.-A.K.); 2 Department of Thoracic Surgery, Wrocław Medical University, Grabiszynska 105, 53-439 Wrocław, Poland; 3 Urology Center Student Scientific Club, Wrocław Medical University, 50-556 Wrocław, Poland

**Keywords:** left upper lobe, lobectomy, segmentectomy, arterial branching pattern, VATS

## Abstract

**Objective**: The arterial anatomy of the left upper lobe exhibits the greatest variability in branching patterns among all pulmonary lobes. This lobe is commonly described as having four segments: the fused apicoposterior segment (S1+2), the anterior segment (S3), and the lingular segments (S4 and S5). Each segment may contain subsegments with distinct vascular supplies. Although several studies have examined patterns and diversity of branching, a comprehensive assessment of the incidence of these variations has not yet been performed. **Methods**: This systematic review was conducted in accordance with a protocol registered in PROSPERO (CRD42024546839). The search was performed between December 2023 and February 2024. A systematic search of databases was carried out to identify publications describing arterial branching patterns supplying the left upper lobe of the lung in adults. Furthermore, we collected and analyzed data on the relationship between the different origins of the lingular arteries and the corresponding bronchial and venous patterns. The AUQA tool was used to perform bias assessment. Data extraction included study characteristics, participant demographics (listed in AUQA), and anatomical variables based on the Yamashita classification of LUL arterial patterns and the number of branches supplying the left upper lobe. **Results**: In total, 15 publications were included (3313 cases). Lobar vasculature was firstly categorized more broadly, analyzing the number of branches from the left pulmonary artery, which supplies the left upper lobe, most commonly four branches. Then, analysis based on the Yamashita classification was performed, and Type A (A3, A(1+2) a+b, A(1+2) c) was established as the most common variant. **Conclusions**: The left upper lobe is most commonly supplied by four different arterial branches, followed by three and five. Differences in arterial branching patterns between Asian and Western populations may represent an important distinguishing factor. According to Yamashita’s classification, Type A (A3, A1+2a+b, A1+2c) is the most frequently observed pattern. Further attention should be directed to the relationship between the presence of a common trunk and the origin of the lingular arteries. Detailed knowledge of this anatomy remains fundamental for segmental thoracic surgery.

## 1. Introduction

Left upper lobe arterial vascularization (LULAV) is commonly recognized as the most variable arterial branching pattern among the lobes of the lung [1]. The growing popularity of video-assisted thoracoscopic surgery (VATS) and increasing frequency of segmental resection have demonstrated that successful segmental resection relies on excellent anatomical knowledge of branching patterns in combination with CT imaging and 3D reconstruction. In contemporary thoracic surgery, VATS accounts for nearly 80% of all lung resections. Prior studies have reported that intraoperative vessel injury during VATS was more frequent during left upper lobectomy and right upper lobectomy in comparison to other pulmonary resections [2,3].

The aim of this review is to establish an evidence base of anatomical data on arterial branching patterns to support advanced segmental resections by facilitating easier identification of the anatomic variant, underlining possible relations between arterial, venous, and bronchial branching patterns. These data may facilitate both preoperative planning and intraoperative decision-making.

The LULAV consists of general arterial branches to all four segments of the left upper lobe. The left upper lobe (LUL) is divided into apicoposterior (S1+2), anterior (S3), superior lingular (S4), and inferior lingular (S5) segments. Subsequently, they are further divided into subsegments with the following nomenclature: a (posterior and superior), b (inferior and anterior), and c (lateral), if present [4,5]. Each of them has an artery corresponding to segmental and subsegmental nomenclature. Previous studies have described variation in the number of branches, ranging from two to seven arteries supplying the LUL. There has been ongoing development in anatomical classification of LUL arterial branching. Over the years, different classification systems have been established. The first anatomical classification for arterial variations was proposed by Richard A. S. Cory and Edward J. Valentine and distinguished 29 types, but 1–4 types accounted for more than 60% of all cases. Type I consists of four separate arterial branches which are ramified from the LPA: the apicoanterior common trunk, apical artery, posterior artery, and common trunk for both lingular arteries. In Type II, only three separate arteries were identified: the apicoanterior trunk, posterior artery and common lingular artery. Type III has four distinct arteries: the apical posterior, apical anterior, posterior and lingular artery. Type IV is represented by the apicoanterior, apical and common trunk for the posterior and lingular arteries [6].

**Figure 1:**
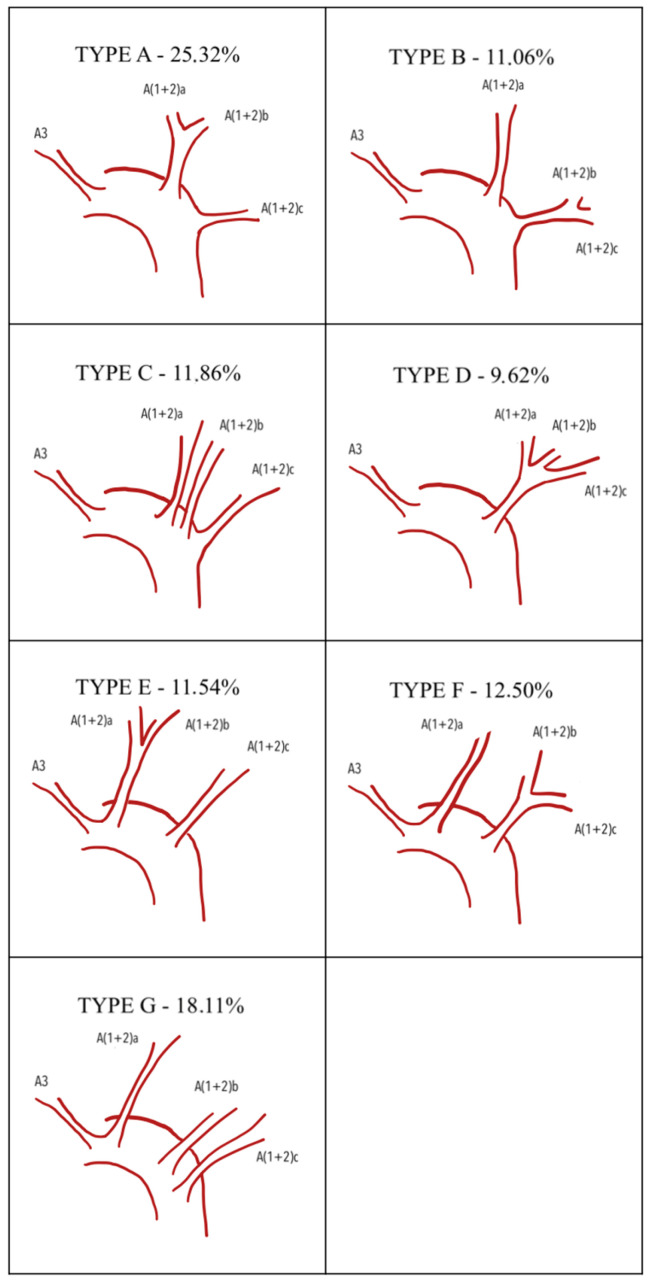
Apicoposterior and anterior artery. A—segmental artery in accordance with segment number; a, b, c—subsegmental branches.

**Figure 2:**
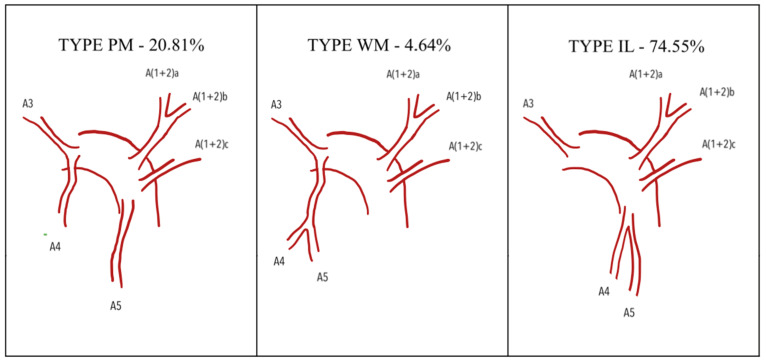
Lingular artery. A—segmental artery in accordance with segment number; a, b, c—subsegmental branches.

Yamashita proposed another classification of the apicoposterior + anterior arterial branches (A1+2+A3), consisting of seven types. When A3 arises independently from A1+2, the types are as follows: Type A (A1+2a+b, A1+2c); Type B (A1+2a, A1+2b+c); Type C (A1+2a, A1+2b, A1+2c); and Type D (A1+2a+b+c). When A3 has a common trunk with A1+2, the types include Type E (A3+A1+2a+b, A1+2c); Type F (A3+A1+2a, A1+2b+c); and Type G (A3+A1+2a, A1+2b, A1+2c) (Figure 1). Lingular arteries were categorized into a mediastinal type (M-type) and an interlobar type (IL-type). The mediastinal type is further divided into a partially mediastinal type (pM-type) and a wholly mediastinal type (wM-type) (Figure 2). Yamashita separated lingular arteries into apicoposterior and anterior segments due to anatomical differences (with the lingula as an equivalent of a middle lobe), surgical approaches to segmental resections, and the relatively rare common origin of lingular arteries with other segments of the LUL [7]. Maciejewski, in 1990 [8], established a classification based on cadaveric lung dissection. He categorized all patterns into four types, divided them specifically into subtypes, and then subdivided the subtypes into variants. In Type I, the lobe was vascularized by the apicoanterior trunk (A1+3) and numerous branches for other segments and subsegments which allow Type I to be categorized into 4 subtypes and 10 variants. Type II is characterized by independent ramification of apicoposterior and anterior segmental arteries from the left pulmonary artery. In this type, two subtypes and eight variants were differentiated. In Type III, the apicoposterior trunk (A1+2) was constant and a variety of other branches allowed for differentiation of three subsegments and nine variants. Type IV consists of an apicoposteroanterior trunk (A1+2+3). The number and configuration of other branches enabled four subtypes and four variants to be distinguished. All types were described in terms of frequency of occurrence, from I (the most frequent) to IV (the least frequent) [8]. More recently, H. Nomori and M. Okada published a book where they propose a more systematic and functional approach to segmentectomy and to major pulmonary resections. They classified arteries supplying each bronchopulmonary segment or subsegment as independent arterial branches arising directly from the LPA. The following arteries were described: A1+2 (apicoposterior), with four alternative branching patterns; A3 (anterior), with a mediastinal and mediastino-interlobar type; A4+5 (lingular arteries), with an interlobar type where A4 and A5 branch separately; an interlobar type with a common A4 and A5 trunk; a mediastinal type, where both arteries branch from the main pulmonary artery; and the last one, a mixed mediastino-interlobar type [9]. All of these types were described in terms of frequency of occurrence, with the most common ones being described first.

The lungs, especially the left upper lobe, are known for variability in anatomic variations, of which the misidentification may result in catastrophic consequences. The identification of the anatomic variant is at the core of the whole planning process. A profound level of knowledge about segmental anatomy of arteries may reduce vascular injury risk during all thoracoscopy interventions, as well as enabling more efficient planning of the entire surgical process.

## 2. Materials and Methods

This systematic review was conducted in accordance with a protocol registered in PROSPERO (CRD42024546839). The PRISMA 2020 checklist is provided in the Supplementary Materials (File S1).

The search strategy was as follows: Major online medical databases such as PubMed, Scopus, Embase, Web of Science, Ebsco, and Google Scholar were searched to find all studies considering the segmental branching pattern of the left upper lobe of the lung. The systematic search was performed in six stages. The search terms listed below were used in all databases. The Google Scholar search was an exception, as we used complete phrases as search terms without the AND connector. We also only reviewed the first 200 results after sorting them by relevance, because this allows for an efficiency similar to that of searching major medical databases in comparison to reviewing all results [10]. The search was performed between December 2023 and February 2024 (January 1945–December 2023).

Search 1: (Lobar branches) AND (pulmonary artery).

Search 2: (left pulmonary artery) AND (CT) AND (variations).

Search 3: (Left pulmonary artery) AND (anatomy) AND left upper lobe).

Search 4: (Left pulmonary artery) AND (branching) AND (left upper lobe).

Search 5: (Left pulmonary artery) AND (anatomy) AND (arterial tree branching) AND (left upper lobe).

Search 6: (Left pulmonary artery) AND (anatomy) AND (branching pattern) AND (variation).

The number of records identified was as follows: Pubmed, 640; Scopus, 450; Embase, 468; Web of Science, 330; Ebsco, 86; Google Scholar, 1200.

Eligibility assessment was conducted as follows: An extraction form was created independently by CK and MA. Initially, a total of 3174 articles were screened independently by two reviewers (KD and SW); if a consensus was not achieved, RT was consulted. Then, extraction was performed by another two independent reviewers (MT and RT). Duplicate records were subsequently removed. Potential risk of bias was assessed by the AUQA (File S2) toolas described in Henry et al. [11]. To minimize bias and maintain methodological rigor, studies such as case reports, case series, conference abstracts, reviews, letters to the editor, and those with incomplete or irrelevant data were excluded. Included studies were required to clearly describe sublobar arterial variations in the left upper lobe, encompassing all segments and subsegments, and to be based on clinical, radiological, intraoperative, or cadaveric research. Studies were excluded if they involved non-human subjects, were written in languages other than English or German, included pediatric or embryonic populations, or did not provide extractable data on the number and branching pattern of arteries supplying the left upper lobe.

The chosen classification was as follows: Out of the variety of different classifications systems for describing LUL arterial variation patterns, we decided to apply the Yamashita classification. This classification was most broadly used in publications we encountered. Furthermore, other articles which did not use this classification were easily convertible to the Yamashita classification. Also, due to its separation of lingular arteries from other segmental arteries of the LUL, this classification can be easily applied during segmental resections.

Extraction: During data extraction, 5 studies were suitable for adjustment to the Yamashita classification. Also, some publications did not distinguish lingual arteries from other arteries supplying the left upper lobe, and one publication did not present the frequency of occurrence of the described types. These included 5 publications that described arterial branching patterns with precise classification of sublobar branches. On this basis, we could apply the Yamashita classification to all the results described in those publications. The total number of patients was 1248. Of these 5 publications, all were based on CT data and 1 was based on combined CT and intraoperative data. CT data were classified after 3D reconstruction. Then, a further 11 articles that traced the number of arteries supplying the left upper lobe were included. These studies could be divided into two groups: earlier research, where data were obtained intraoperatively or through cadaveric dissection, with one outlier, Maciejewski et al. [8], in which the LPA and the bronchus were injected with a 65% solution of Duracryl (Spofa-Dental) and then digested in sulfuric acid; and modern studies, which used CT and 3D reconstruction. Data extraction included study characteristics, participant demographics (listed in AUQA), and anatomical variables based on the Yamashita classification of LUL arterial patterns and the number of branches supplying the left upper lobe. For missing data, authors were contacted at first; if this was unsuccessful, we proceeded with the available data. All ambiguities were resolved through consensus between the reviewers or consultation with a senior expert (RT).

Outcome analysis was conducted as follows: The aim of the extraction was to identify the distinct number of branches supplying the left upper lobe, including both the lingula and the apical segment. Additionally, another objective was to determine the exact frequency of subsegmental arterial patterns according to the aforementioned Yamashita classification.

## 3. Results

Initially, the database search identified a total of 144 publications. After removing 120 duplicates, 24 records remained for screening. Subsequently, six studies were excluded due to impossible data extraction, and three publications were excluded because they did not provide the frequency of occurrence of the vascularization patterns described in the text.

Overall, 15 publications met the inclusion criteria and were incorporated into this review (Table 1), comprising 3313 cases in total. Lobar vasculature was initially categorized using a broad approach by analyzing the number of branches arising from the left pulmonary artery supplying the left upper lobe. In this dataset, the most commonly observed configuration consisted of four branches. We analyzed cases from 11 different publications to trace the number of branches to the left upper lobe. Three of the studies were conducted in the USA, two in Poland and China, and one each in Jamaica, Russia, and Japan. The total number of patients from all the selected publications was 1979 (Table 2). The numbers of branches to the left upper lobe varied from one to seven. Most commonly, there were four (44.16%) branches, then three (25.54%), then five (19.05%), and then six (4.40%) and two (3.54%). A single branch to the left upper lobe was reported only once, by Milloy et al. (1968) [12], so we can treat it as an anomaly. Also, five publications were analyzed to trace the frequency of sublobar and subsegmental arteries. We used the Yamashita classification. Four studies were conducted in China and one in Japan. In total, they included 1248 patients with A3 and A1+2 branching types and 1169 patients with lingular branching types. These findings suggest that the frequency of these patterns may differ in Western populations. The most common pattern was Type A (25.32%), followed by Type G (18.11%) and Type F (12.5%). Types B, C, and E occurred at similar frequencies—11.06%, 11.86%, and 11.54%, respectively. Type D was the least frequent.

For lingular arterial branching, the most common pattern was the interlobar type, observed in 74.55% of cases.

For lingular arterial branching, the most common pattern was the interlobar type, observed in 74.55% of cases. The mediastinal type (M-type) was present in 25.45% of cases, with 20.81% of the cases classified as partially mediastinal (pM-type) and 4.64% as wholly mediastinal (Table 3).

## 4. Discussion

Arterial branching patterns in the left upper lobe hold significant value for thoracic surgeons during upper lobe resection—both lobectomy and segmentectomy. Recently, we have observed an increasing number of segmental resections for tumors smaller than 2 cm in diameter with pathologically confirmed node-negative disease in the hilar and mediastinal lymph nodes. These procedures have been shown to be non-inferior in comparison to lobar resection and allow for lesser impairment of pulmonary functions [23]. Increasing VATS and RATS segmental and even subsegmental resection require precise anatomic data, which are crucial for safe and sufficient resections. We aimed to provide quality data and present a new, more systematic approach for collection of all anatomical branching patterns, especially arterial ones. A summary of data from all included publications indicates that four arterial branches represent the most frequent variant. Among the 11 publications analyzed, eight highlighted four arterial branches as the most common variant, while three reported three branches as the most frequent. Regarding the second most common variant, five studies identified three branches, three studies reported four branches, and another three studies reported five branches. We tried to perform a statistical analysis to evaluate any disparities between Western and Asian populations, but the data turned out to be inadequate for such an analysis. Further studies should be encouraged to develop better worldwide standards of care. The most common type of branching, according to our analysis, was type A of the Yamashita classification, present in 316 cases, which was 25.32% of all cases. Three studies also recognized Type A as the most common pattern, one study reported Type C as most frequent, and another study found Type E to be the predominant type (Table 3, Figure 1). Interlobar origin of the lingular arteries was the most frequently observed pattern, identified in 867 cases (74.55%) (Table 2, Figure 2). However, Youjun Deng et al. (2022) found no cases with presence of a common trunk for A3 and A(1+2) arteries. Furthermore, they established another subdivision for left upper lobe arterial branching when lingular arteries crossed the area of S3 and S1+2 [22]. Data provided by Hao Xu et al. (2023) enabled us to classify their arterial branching patterns into only Yamashita types E and G. Together, these two types accounted for 81% of all described patterns, suggesting that the remaining types may represent anatomical anomalies [21]. Among all the articles, three described an anomalous origin of lingular artery A5 from the basilar artery. Two studies each reported one case of this anomalous pattern [16,24]. Another reported the occurrence of at least a subsegmental lingular branch from the basal artery in 21% of cases. Therefore, we conclude that further studies are required to establish an accurate frequency of occurrence of these arterial branching anomalies. The association established in previous publications between venous vascular patterns and lingular artery origin, as well as the correlation between the type of bronchial branching pattern and lingular artery, was also reviewed. However, there were insufficient publications to carry out a systematic review of this subject. The studies conducted by Gao et al. and Li Z et al. should be used as a model in exploring these correlations [20,25].

**Figure 3:**
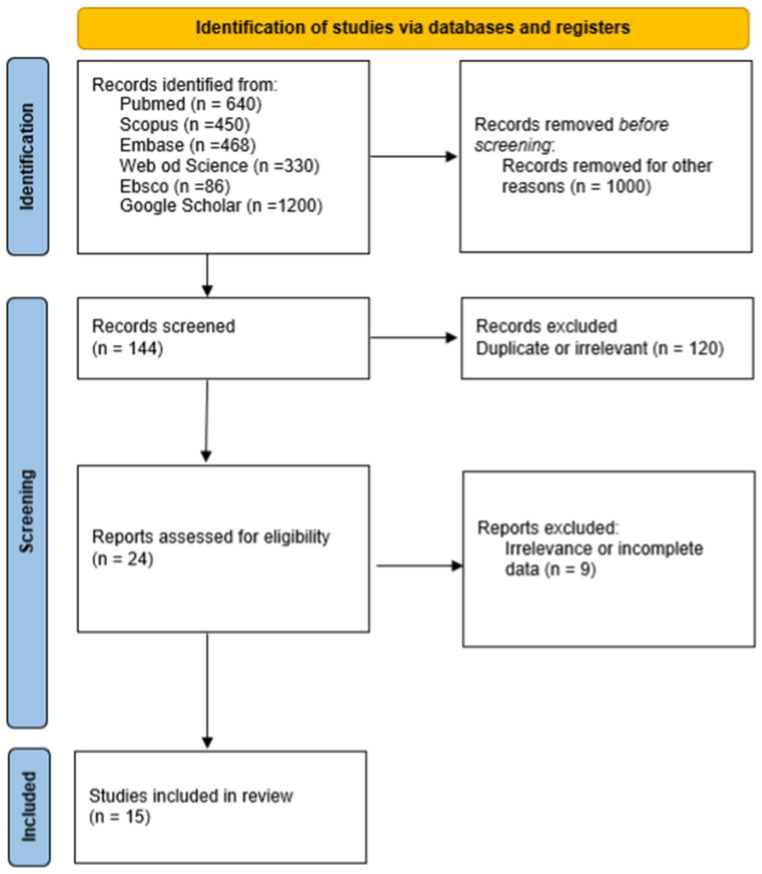
PRISMA flow chart of included studies.

During data collection, we identified a potential correlation between the presence of a common trunk for A(1+2), A3 and the origin of the lingual artery (Figure 2 and Figure 3). This information could be potentially crucial in intraoperative decision-making. We are currently working on the development of such a study. These associations may increase awareness of different vascular branching during parenchymal dissection, especially useful during left upper lobectomy. However, most of the studies were conducted in Asia, and the results might have been different in other populations. To establish more valuable data, we could create a platform for reporting all branching patterns, especially for cases when 3D reconstruction was done preoperatively. If sufficient data were provided, creation of bronchovascular patterns for segmental and subsegmental resections could be created. This could be crucial for successful subsegmental resection, because the main problem is still operative time, and blood loss is higher in comparison to segmental resections [20].

In this review, we aimed to explore and utilize the available quantitative data. Although the data were too heterogeneous and insufficient to support a full meta-analysis, they allowed us to outline several potentially relevant directions for future research. Summarizing numerical findings from different centers enabled a few indicative analyses. These results are not a formal component of this review and are intended for guidance only; however, they highlight notable relationships that warrant further comprehensive investigation.

The preliminary analysis suggests that patterns of venous vascularization of the LUL can vary between Asian and Western populations, which indicates the anatomical variance as well as methodological differences between analyzed studies. Similar observations are made regarding a possible relationship between the origin of the lingular artery and the course of the venous outflow tracts, as well as between the origin of the arteries and the presence of a common trunk for A3 and A(1+2) and the configuration of the bronchial division of the left upper lobe segments.

Although statistically significant in preliminary analyses, these relationships are based on data that originate from a very limited number of publications and are susceptible to errors resulting from differences in anatomical definitions, research techniques, and reporting methods. Therefore, these results should be interpreted only as preliminary research that may inspire further, more precise morphometric analyses or future meta-analyses based on homogenic material. In particular, conducting comparative studies in large, well-defined populations with clear criteria of venous and arterial vascularization of the left upper lobe should be considered. This approach is essential to confirm or refute the conclusions from this review.

Although the results presented here are only preliminary, they highlight a clear need for further research into the relationships among the venous, arterial, and bronchial systems of the left upper lobe, which may have important clinical and anatomical implications.

## 5. Conclusions

Disparities between Western and Asian populations in the context of the number of arteries which supply the LUL deserve further attention, especially in the context of creating more applicable standards of care across the globe.

The gathered data might suggest that three arterial branches are more commonly recognized in Western populations, while four and five branches are more commonly recognized in Asian populations. Type A (A3, A(1+2) a+b, A(1+2) c) was established as the most common, and in general, variants where the common trunk for A3 and A(1+2) was absent were slightly more frequent. The possible correlation between presence of a common trunk and the origin of the lingular artery warrants further in-depth analysis.

## Figures and Tables

**Table 1:** Characteristics of the studies included in the review.

References	Location	Sample Size (Male, Female, Race)	Age (Years)	Method of Evaluation of Arteries
Barrett et al., 1956 [13]	USA	100	N/A	Dissection
Boyden, 1946 [14]	USA	50	N/A	Dissection
Cory and Valentine, 1959 [6]	Jamaica	Jamaican, 426	N/A	Dissection
Milloy et al., 1968 [12]	Jamaica	300 (not specified)	N/A	Dissection
Molchanow, 1958 [8]	USSR	100 (not specified)	N/A	Dissection
Skorupka and Topol, 1979 [15]	Poland	40	N/A	Dissection
Maciejewski, 1990 [8]	Poland	European, 100	16–80	The LPA and the bronchus were injected with a 65% solution of Duracryl (Spofa-Dental) and then digested in sulfuric acid
Fang et al. 2021 [16]	China	76 males 60 females Asian, 136	18–75	CT—Angiography
Xiaochao Ma et al., 2023 [17]	China	216 males 204 females Asian, 420	56.54 ± 10.08 males 56.12 ± 9.55 females	CT—Angiography
Alex Fourdrain et al., 2018 [18]	France	25 males 19 females European, 44	N/A	CT—Angiography
Ryunosuke Maki et al. 2022 [19]	Japan	Asian, 539	N/A	CT—Angiography
Gao C et al. 2023 [20]	China	Asian, 151	N/A	3-DCTBA
Xu H et al. 2023 [21]	China	Asian, 100	N/A	CT
Deng Y et al. 2023 [22]	China	39 males 64 females Asian, 103	28–78 years, with a median age of 55 years	CT
Gao C et al. 2021 [1]	China	Asian, 404	N/A	3-DCTBA

**Table 2:** Numbers of branches from PA supplying the left upper lobe.

Reference	Number of Cases	1	2	3	4	5	6	7	Country
Barrett et al., 1956 [13]	100	0	5	31	41	15	7	1	USA
Boyden, 1946 [14]	50	0	0	9	20	14	6	1	USA
Cory and Valentine, 1959 [6]	150	0	10	36	76	20	6	2	Jamaica
Milloy et al., 1968 [12]	300	1	15	138	107	36	3	0	USA
Molchanow, 1958 [8]	100	0	6	52	39	1	1	1	Russia
Skorupka and Topol, 1979 [15]	40	0	2	14	21	3	0	0	Poland
Maciejewski, 1990 [8]	100	0	2	26	49	19	3	1	Poland
Fan K, 2022 [16]	136	0	24	60	44	8	0	0	China
Xiaochao Ma, 2023 [17]	420	0	6	107	212	91	2	2	China
Alex Fourdrain, 2018 [18]	44	0	0	10	22	10	2	0	France
Ryunosuke Maki, 2022 [19]	539	0	0	23	243	160	57	0	Japan
Total	1979	1	correct70	506	874	377	87	8	
Percentage	100%	0.05%	3.54%	25.57%	44.16%	19.05%	4.40%	0.40%	

**Table 3:** Occurrence of different types of arterial branching according to Yamashita classification. A—segmental artery in accordance with segment number; a, b, c—subsegmental branches.

Type	Description	Chuan Gao, 2023 [20]	Youjun Deng 2022 [22]	Ryunosuke Maki, 2022 [19]	Chuan Gao, 2021 [1]	Hao Xu 2023 [21]	Sum	Percentage
A	A(1+2)a+b, A(1+2)c, A3	31	33	143	109	0	316	25.32%
B	A(1+2)a, A(1+2)b+c, A3	28	21	61	28	0	138	11.06%
C	A(1+2)a, A(1+2)b, A(1+2)c, A3	11	35	71	31	0	148	11.86%
D	A(1+2)a+b+c, A3	23	14	30	53	0	120	9.62%
E	A3+A(1+2)a+b, A(1+2)c	22	0	27	49	46	144	11.54%
F	A3+A(1+2)a, A(1+2)b+c	29	0	59	68	0	156	12.50%
G	A3+A1+2a, A1+2b, A1+2c	25	0	100	66	35	226	18.11%
		169	103	491	404	81	1248	
pm	pM type (A4 from mediastinum A5 interlobar)	32	17	109	84	0	242	20.81%
wm	wM type (A5 and A5 from mediastinum)	6	7	18	23	0	54	4.64%
il	IL type (A4 and A5 from interlobar part)	113	79	378	297	0	867	74.55%
		151	103	505	404	0	1163	

## Data Availability

No new data were created or analyzed in this study.

## Supplementary Materials

The following supporting information can be downloaded at https://www.mdpi.com/article/10.3390/jcm15051724/s1, File S1: PRISMA 2020 Checklist; File S2: AQUA LUL (Bias Assessment).
