# Supplementary material for: Arterial Branching Patterns Supplying the Left Upper Lobe of the Lung and Their Incidence: A Systematic Review
Source: J Clin Med. 2026 Feb 25;15(5):1724. doi: 10.3390/jcm15051724 (PMC12985616; doi:10.3390/jcm15051724)
Supplement: Supplementary file 1 [file jcm-15-01724-s001.zip › jcm-4131988-supplementary.pdf]

File S1: PRISMA 2020 Checklist

Item #	Checklist Item	Location in Manuscript (Section, Table, or Quote)
1	**Title**	Title Page: "A systematic review of arterial branching patterns..."
2	**Abstract**	Abstract section
3	**Rationale**	Introduction, paragraphs 1–3: Discusses anatomical variability and its clinical significance in VATS
4	**Objectives**	Introduction, end of paragraph 3: "The aim of this review is to establish an evidence base..."
5	**Eligibility criteria**	Materials and Methods, "Eligibility assessment": Criteria for inclusion (clinical, radiological studies) and exclusion (pediatric, non-human, etc.)
6	**Information sources**	Materials and Methods, "Search strategy": Lists PubMed, Scopus, etc. searched from Dec 2023 to Feb 2024
7	**Search strategy**	Materials and Methods, "Search strategy": Includes 6 detailed search query strings
8	**Selection process**	Materials and Methods, "Eligibility assessment": Describes 2 independent reviewers and consultation.
9	**Data collection**	Materials and Methods, "Extraction" and "Eligibility assessment": Outlines how MT, RT, CK, and MA handled data
10a	**Data items**	Materials and Methods, "Extraction": Lists demographics, anatomical variables (Yamashita classification), and branch counts
10b	**Data items**	Materials and Methods, "Extraction": Mentions contacting authors for missing data
11	**Study risk of bias**	Materials and Methods, "Eligibility assessment": AQUA supplementary materials.
12	**Effect measures**	N/A (This review reports anatomical frequencies and percentages rather than clinical effect measures like Odds Ratios)
13a-e	**Synthesis methods**	Materials and Methods, "Chosen classification" and "Outcome analysis": AQUA supplementary materials.
14	**Reporting bias**	N/A (Not performed due to data heterogeneity)
15	**Certainty assessment**	N/A (No formal GRADE assessment was performed, results are noted as "indicative")
16a	**Study selection**	Results, paragraph 1: 144 identified, 15 finally included ; also Figure 3 (Prisma flowchart)
17	**Characteristics**	Results, paragraph 3: Summary of 15 publications and 3313 cases; Detailed in Table 1
18	**Bias in studies**	Mentioned as procedure in Materials and Methods. AQUA supplementary materials.
19	**Individual results**	Table 2: Number of branches per study; Table 3: Yamashita types per study
20a-d	**Synthesis results**	Results, a- AQUA supplementary materials. paragraphs 4–11: Summary statistics (44.16% have 4 branches, Type A is 25.32%)
21	**Reporting bias**	AQUA supplementary materials.
22	**Certainty results**	N/A
23a	**Discussion (results)**	Discussion, paragraphs 1–4: Summary of primary variants.
23b	**Discussion (limits)**	Discussion, paragraphs 6, 10, and 11: Notes data heterogeneity, limited publications, and Asian population bias
23c	**Discussion (interpret)**	Discussion, paragraphs 1–2: Clinical implications for VATS/RATS
24	**Registration**	Abstract and Materials and Methods: PROSPERO (CRD42024546839)
25	**Support**	Funding section: "This research received no external funding"
26	**Conflicts**	Conflicts of Interest: "The authors declare no conflicts of interest"
27	**Data availability**	Data Availability Statement: (Currently blank in manuscript, but data is present in tables)

File S2, AQUA LUL(Bias Assessment)

1. **Study bias assessment**

	A1	A2	A3	A4	A5	A6	A7	A8	A9	A10	A11	A12	A13	A14	A15	A16	A17	A18	A19	A20	A21	A22	A23	A24	A25	A26	A27	A28	A29
Barrett et al., 1956 **[12]**	+	+	+	-	+	-	+	-	-	+	+	+	+	+	-	-	+	-	-	-	-	+	-	-	-	-	-	-	-
Boyden, 1946 **[13]**	+	+	+	+	-	-	+	-	-	+	+	+	+	-	-	-	+	-	-	-	-	+	-	-	-	-	-	-	-
Cory and Valentine, 1959 **[6]**	+	+	+	+	-	-	+	-	-	+	+	+	+	-	-	-	+	-	-	-	-	+	-	-	-	-	-	-	-
Milloy et al., 1968 **[14]**	+	+	+	+	-	-	+	-	-	+	-	+	+	-	-	-	+	-	-	-	-	+	-	-	-	-	-	-	-
Molchanow, 1958 **[8]**	+	+	+	+	-	-	+	-	-	+	-	-	+	-	-	-	+	-	-	-	-	+	-	-	-	-	-	-	-
Skorupka and Topol, 1979 **[15]**	+	+	+	+	-	-	+	-	-	+	-	-	+	-	-	-	+	-	-	-	-	+	-	-	-	-	-	-	-
Maciejewski, 1990 **[8]**	+	+	+	+	-	-	+	-	-	+	-	+	+	-	-	-	+	-	-	-	-	+	-	-	-	-	-	-	-
Fang et al. 2021 **[16]**	+	+	+	+	+	+	+	+	+	+	+	+	+	+	+	+	+	+	+	-	+	+	+	+	+	+	+	+	+
Xiaochao Ma et al., 2023 **[17]**	+	+	+	+	+	+	+	+	+	+	+	+	+	+	+	+	+	+	+	-	+	+	+	+	+	+	+	+	+
Alex Fourdrain et al., 2018 **[18]**	+	+	+	+	+	+	+	+	+	+	+	+	+	+	+	+	+	+	+	-	+	+	+	+	+	+	+	+	+
Ryunosuke Maki et al. 2022 **[19]**	+	+	+	+	+	+	+	+	+	+	+	+	+	+	+	+	+	+	+	-	+	+	+	+	+	+	+	+	+
Gao C et al. 2023 **[25]**	+	+	+	+	+	+	+	+	+	+	+	+	+	+	+	+	+	+	+	-	+	+	+	+	+	+	+	+	+
Xu H et al. 2023 **[22]**	+	+	+	+	+	+	+	+	+	+	+	+	+	+	+	+	+	+	+	-	+	+	+	+	+	+	+	+	+
Deng Y et al. 2023 **[21]**	+	+	+	+	+	+	+	+	+	+	+	+	+	+	+	+	+	+	+	-	+	+	+	+	+	+	+	+	+
Gao C et al. 2021 **[1]**	+	+	+	+	+	+	+	+	+	+	+	+	+	+	+	+	+	+	+	-	+	+	+	+	+	+	+	+	+

The outcomes of study bias assessment are shown in the form of a table, where each item from AQUA tool was evaluated for every included study. “+” means that specific study fulfils item criteria, whereas “-” means it does not.
